# Two Sides of the Same Coin: The Roles of KLF6 in Physiology and Pathophysiology

**DOI:** 10.3390/biom10101378

**Published:** 2020-09-28

**Authors:** Saiful E. Syafruddin, M. Aiman Mohtar, Wan Fahmi Wan Mohamad Nazarie, Teck Yew Low

**Affiliations:** 1 UKM Medical Molecular Biology Institute (UMBI), Universiti Kebangsaan Malaysia, Cheras, Kuala Lumpur 56000, Malaysia; m.aimanmohtar@ppukm.ukm.edu.my (M.A.M.); lowteckyew@ppukm.ukm.edu.my (T.Y.L.); 2 Biotechnology Programme, Faculty of Science and Natural Resources, Universiti Malaysia Sabah, Kota Kinabalu 88400, Malaysia; wanfahmi@ums.edu.my

**Keywords:** transcription factor, gene regulation, developmental process, inflammation, cancer, KLFs

## Abstract

The Krüppel-like factors (KLFs) family of proteins control several key biological processes that include proliferation, differentiation, metabolism, apoptosis and inflammation. Dysregulation of KLF functions have been shown to disrupt cellular homeostasis and contribute to disease development. KLF6 is a relevant example; a range of functional and expression assays suggested that the dysregulation of KLF6 contributes to the onset of cancer, inflammation-associated diseases as well as cardiovascular diseases. KLF6 expression is either suppressed or elevated depending on the disease, and this is largely due to alternative splicing events producing KLF6 isoforms with specialised functions. Hence, the aim of this review is to discuss the known aspects of KLF6 biology that covers the gene and protein architecture, gene regulation, post-translational modifications and functions of KLF6 in health and diseases. We put special emphasis on the equivocal roles of its full-length and spliced variants. We also deliberate on the therapeutic strategies of KLF6 and its associated signalling pathways. Finally, we provide compelling basic and clinical questions to enhance the knowledge and research on elucidating the roles of KLF6 in physiological and pathophysiological processes.

## 1. Introduction

The human KLF genes encode Krüppel-like factors, a family of zinc finger DNA-binding proteins. These KLFs are the mammalian homologs of Krüppel, a *D. melanogaster* gene that is evolutionarily conserved across species [1]. In fruit flies, mutations in the Krüppel gene were reported to disrupt the anterior-posterior segmentation pattern during the early stage of embryogenesis [2]. These seminal findings by Nüsslein-Volhard and Wieschaus, and their subsequent works in understanding the genetic determinants of early embryonic development, led them to the 1995 Nobel Prize in Physiology, along with Edward B. Lewis. Within the KLF family, EKLF or KLF1, which is an important transcription factor that regulates erythroid development and maturation [3], was the first member of the family discovered in humans. The deletion of KLF1 in mice has been demonstrated to lead to anaemia and β-globin deficiency and is therefore embryonically lethal [4]. The discovery of this erythroid-specific KLF1 transcriptional regulator was ensued by the identification of 17 additional members of this family, designated as KLF2-KLF18. Nonetheless, KLF18, which is the latest member of this family, is classified as a pseudogene that has arisen from gene duplication or retro-transposition events [5].

The human KLFs family has been shown to be involved in regulating a myriad of cellular processes, including proliferation, differentiation, metabolism and pluripotency maintenance, as well as inflammation and injury responses [1]. Therefore, the dysregulation in KLF functions is expected to disrupt cellular homeostasis and can consequently result in the development of diseases [1]. In this review, we focus on KLF6. The review first starts with the current knowledge of the KLF6 gene and the protein structures. Then, we comprehensively discuss the roles of KLF6 in regulating normal physiological processes, as well as its involvement in driving the pathogenesis of several diseases, with specific emphasis on the opposing roles of full-length KLF6 and KLF6-SV1. In addition, we also posit KLF6 therapeutic potentials. Last, we provide several compelling fundamental and clinical questions that need to be addressed to improve our overall understanding of KLF6 functions in health and diseases.

## 2. KLF6 Gene Structure and Post-Transcriptional Modifications

**Figure 1:**
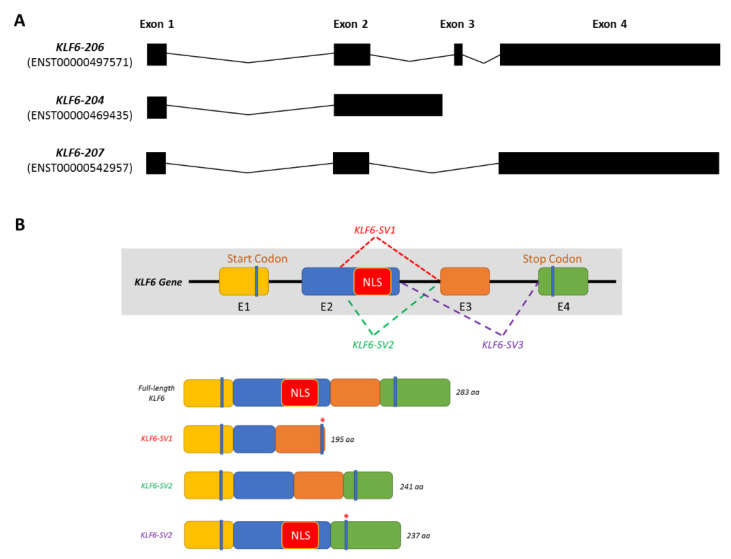
KLF6 gene structure and the associated post-transcriptional modifications. (**A**) The KLF6 gene can be transcribed to produce 7 different transcripts, of which only KLF6-204, KLF6-206 and KLF6-207 are translated into proteins. (**B**) The primary KLF6 transcript, KLF6-206, contains four exons whereby germline mutation IVS 1-27 G > A can induce alternative splicing that gives rise to three additional KLF6 spliced variants: KLF6-SV1, KLF6-SV2 and KLF6-SV3.

The human KLF6 gene is located on the short arm of chromosome 10 (10p15). Comprising four exons, the KLF6 gene can be transcribed into seven different transcripts. However, only three transcripts are suggested to be translated into proteins: KLF6-204, KLF6-206 and KLF6-207, as shown in Figure 1A. KLF6-206 (referred to as KLF6 hereinafter) is annotated as the primary transcript, which gives rise to a protein that is 283 amino acids in length. KLF6 was originally identified as a core promoter-binding protein (CPBP) that was highly enriched in the placenta to regulate the expression of pregnancy-specific glycoprotein genes [6,7]. Other than the placenta, the KLF6 gene was also detected and cloned from the liver [8] and leukocytes [9]. In addition to CPBP, several aliases have been assigned to KLF6, including ZF9, the core promoter element-binding protein (COPEB), B-cell-derived protein 1 (BCD1) and the suppressor of tumorigenicity 12 (ST12). KLF6 is involved in modulating the expression of cellular injury response and tissue repair-related genes such as transforming growth factor beta 1 (TGFβ1) and transforming growth factor beta receptor (TGFβR) [10], collagen type 1 alpha 1 (COL1A1) [11], plasminogen activator urokinase (PLAU) [12] and endoglin (ENG) [13].

Meanwhile, a KLF6 G > A germline mutation has been reported to occur at the intervening sequence 1-27 (IVS 1-27 G > A). This single nucleotide polymorphism (SNP) generates de novo splicing sites, resulting in the emergence of three additional KLF6 spliced variants: KLF6-SV1, KLF6-SV2 and KLF6-SV3 [14]. These KLF6 spliced variants differ in length and structure due to the utilisation of different splicing donor and/or splicing acceptor sites (Figure 1B). Directed by the nuclear localisation sequences (NLS) located at the end of exon 2, the full-length KLF6 localises in the nucleus, corroborating its roles as a transcriptional regulator. However, due to alternative splicing, this NLS domain is lost in KLF6-SV1 and KLF6-SV2. Hence, rather than being transported into the nucleus, KLF6-SV1 and KLF6-SV2 are localised in the cytoplasm and are, therefore, unable to regulate the gene transcription process [14]. KLF6-SV3, on the other hand, retains the NLS domain but loses the whole exon 3. Among these three spliced variants, KLF6-SV1 is widely studied, especially in cancer, whereas the roles of the other two isoforms remain elusive [14,15].

## 3. KLF6 Protein Structures

**Figure 2:**
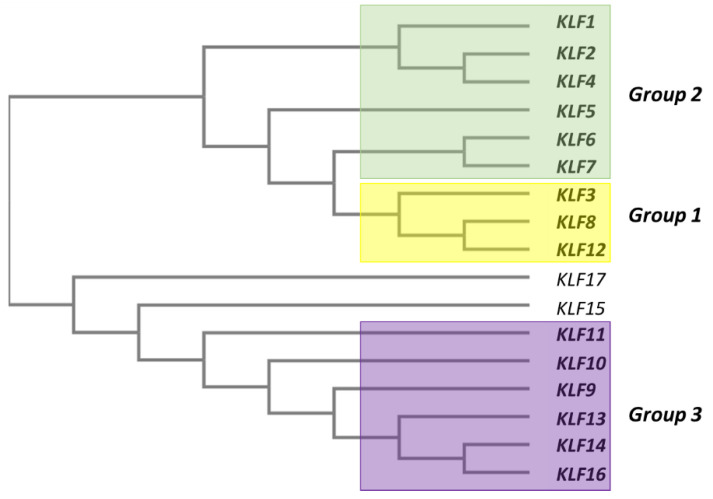
A human KLF family phylogenetic tree generated using the Clustal Omega tool (https://www.ebi.ac.uk/Tools/msa/clustalo/). There are 17 members of the human KLF family, which can be further classified into three subgroups. KLF7 is the closest to KLF6 in the phylogeny.

All human KLF proteins contain three repeats of Cys2-His2 (C2H2) that constitute a high-conserved, DNA-binding zinc finger in the C-terminus. These repeats preferentially bind to the “GC-box” or “CACCC” motifs that normally exist in promoter regions [16]. On the other hand, the N-terminus domain for KLF proteins allow them to interact with different client proteins, including transcription factors, coactivators and corepressors, as well as the chromatin-modifying enzymes [16]. Therefore, the functional diversity of KLFs is determined by their N-terminal domain. The phylogenetic classification of the human KLFs separate these proteins into three distinct subgroups (Figure 2). Group 1 consists of KLF3, KLF8 and KLF12, which function as transcriptional repressors by interacting with the C-terminal-binding proteins (CtBP1/2). KLF1, KLF2, KLF4, KLF5, KLF6 and KLF7, on the other hand, belong to group 2, and they predominantly function as transcriptional activators. Finally, KLF9, KLF10, KLF11, KLF13, KLF14 and KLF16 are in group 3, and they are classified as transcriptional repressors due to their interactions with corepressor Sin3a [1].

**Figure 3:**
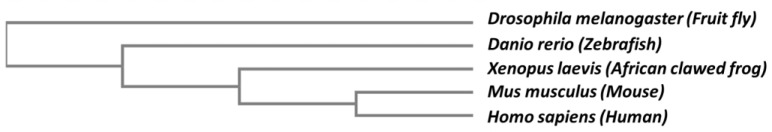
The phylogenetic relationship among the five selected KLF6 orthologs from *D. melanogaster*, *D. rerio*, *X. laevis*, *M. musculus* and *H. sapiens*. The phylogram was generated using the Clustal Omega tool (https://www.ebi.ac.uk/Tools/msa/clustalo/).

**Figure 4:**
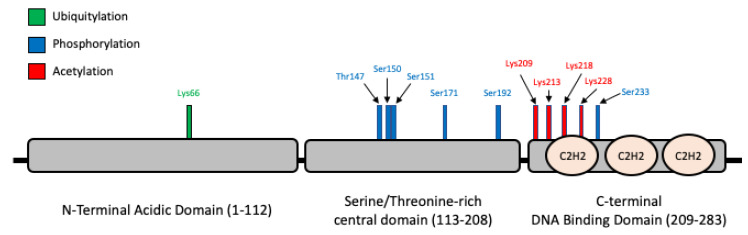
KLF6 protein structure and the associated post-translational modifications. The KLF6 protein consists of three domains: (I) N-terminal acidic domain, (II) serine/threonine-rich central domain and (III) C-terminal DNA-binding domain. The protein structure is adapted from Andreoli et al. [17]. According to PhosphositePlus (https://www.phosphosite.org/), the KLF6 protein undergoes three types of post-translational modifications (PTMs). The sole ubiquitylation site for KLF6 is located at Lys66-Ub. Phosphorylation is the best-documented KLF6 PTM, encompassing T147-p, S150-p, S151-p, S171-p, S192-p and S233-p. Finally, four lysine acetylation sites have been documented for the C-terminal, i.e., S209-Ac, S213-Ac, S218-Ac and S228-Ac.

Due to their close structural and functional characteristics within the human Krüppel-like family, KLF6 and KLF7 are classified together in the same phylogenetic group (Figure 2), whereby a common progenitor has been traced to a gene named LUNA in Drosophila [17,18]. The phylogenetic tree of KLF6 orthologs in several other species is shown in Figure 3. Structurally, KLF6 possesses three functional domains that endow them the ability to bind to specific DNA sequences, localise into the nucleus and regulate the transcription of its downstream targets (Figure 4). The N-terminal acidic domain of KLF6 stretches from Met1 to Ser112. This domain is responsible for recruiting and interacting with other transcription factors and cofactors, such as Sp1, KLF4, p53, hypoxia-inducible factor 1 alpha (HIF1α), runt related transcription factor 1 (RUNX1), E2F1, GTF3C1 and histone deacetylase 3 (HDAC3), to either activate or repress the transcription process in a context-dependent manner [19,20,21,22,23,24,25]. It is noteworthy to point out that sequences in this domain are highly variable among the KLF family members, providing another layer of diversity that may contribute to a wide range of regulatory activities [17,26].

On the other hand, the central domain of KLF6 starts at Ser113 and continues up to Arg208. This central domain is particularly enriched in Ser and Thr residues, rendering this domain a potential target for post-translational modifications, especially phosphorylation. Indeed, it has been demonstrated earlier by Slavin et al. that KLF6 is constitutively phosphorylated in vivo [27]. A number of kinases have been demonstrated to increase the level of phosphorylation of KLF6, although the exact sites of phosphorylation have not been confirmed. For example, Lang et al. reported the increased phosphorylation of KLF6 based on in-vitro and in-vivo ^32^P incorporation assays upon the transient transfection of glycogen synthase kinase 3 beta (GSK3β) and the subsequent transactivation of a p21 [28]. Besides, the phosphorylation of KLF6 by ribosomal protein S6 kinase beta-1 (S6K1) has also been linked to the induction of TGFB gene transcription [29].

The C-terminal domain, Lys209 to Leu283, contains three C2H2 zinc finger-type structures. The NLS precede these C2H2 zinc fingers. The C2H2 zinc fingers are among the ancient and common domains found in the transcription factors of higher eukaryotes [30]. Typically, each C2H2 zinc finger motif is comprised of 25–30 amino acid residues [31]. In the case of KLF6, as well as other KLFs, the first two zinc finger motifs consist of 23 amino acids, and the third zinc finger is 21 amino acids long. These motifs bind the DNA, especially to the GC box or the 5′-CACCC-3′ regions [31,32]. Meanwhile, the three zinc finger motifs are connected by a linker that is highly conserved with a sequence motif (TGE(R/K)(P/k/r)(F/y)X)) and is capable of binding certain other zinc finger proteins with high affinity [32]. Although the exact 3D structure of the KLF6 protein has not been solved, the three C2H2 motifs in the KLF family generally form β-hairpin starting from the N-terminal, followed by α-helix, which form a left-handed ββα structure. Apart from that, the zinc-finger structure is stabilised by the coordination of a zinc atom with two conserved cysteine residues at one end of the β sheet and with two conserved His residues at the α-helix C-terminus. The C2H2 motifs constitute a defining feature not only for the 17 human KLF family members but, also, the related specificity proteins (SPs) transcription factors, whereby more than 65% sequence identity has been demonstrated among this family members [26]. In fact, the specificity proteins (SPs) and KLFs are members of the same higher-rank family of transcription factors, the so-called Sp/KLF family, that share conserved zinc finger domains. These transcription factors are expressed in a tissue-specific manner, and so are their activities and functions. It has been demonstrated that the Sp/KLF family regulates the growth, development, differentiation, proliferation and embryogenesis

## 4. KLF6 Post-Translational Modifications

Post-translational modifications (PTMs) refer to covalent moieties that are added on the amino acid residues to modify, fine-tune and diversify the inherent biological functions of a protein. The KLF6 amino acid residues that undergo PTMs are shown in Figure 4. According to PhosphositePlus, one of the most comprehensive repository for PTMs, a total of six phosphorylation sites have been reported for KLF6 so far, whereby five of them: Thr147-p, Ser150-p, Ser151-p, Ser171-p and Ser192-p, except for Ser233-p, are located at the Ser- and Thr-rich central domain [33,34]. Since all these phosphorylation sites were derived from large-scale phosphoproteomics studies, the precise functional implications for each site has not been systematically investigated, despite that a selected number of kinases that are associated with KLF6 functions have been reported [29,35].

Moreover, there are four Lys acetylation sites reported for KLF6: Lys209-Ac, Lys213-Ac, Lys218-Ac and Lys228-Ac [34,36]. Among these four sites, the first three were discovered by Li et al. by incubating four synthetic peptide fragments that covered the majority of the lysine residues of KLF6 with CREB-binding protein (CBP) and P300/CBP-associated factor (PCAF), followed by an in vitro histone acetyltransferase assay using 3H-labeled acetyl-CoA. As a result, CBP, but not PCAF, was found to acetylate Lys209, Lys213 and Lys218 [36]. The authors further demonstrated that, when Lys209 was mutated to arginine, acetylation at this site was abrogated, leading to the loss of ability of KLF6 to transactivate p21. Interestingly, only one ubiquitylation site has been reported for KLF6 to date, Lys66-Ub, albeit this site was reported by three independent large-scale ubiquitome studies [37,38,39]. This is consistent with the study of Banck et al., who demonstrated that KLF6 could be subjected to proteasome-mediated degradation [40]. Recently, SCFFbxw7 was found to ubiquitylate KLF7 for proteasomal degradation in a manner that was dependent on GSK3β-mediated phosphorylation [41]. Despite KLF7 being the closet paralog of KLF6, the authors’ results showed that only KLF7, but not KLF6, was a bona fide substrate of SCFFbxw7.

## 5. KLF6 Roles in Normal Physiological Processes

### 5.1. Cellular Differentiation and Proliferation

Eukaryotic development is a finely tuned process, orchestrated by the spatiotemporal expression and interactions of developmental-associated transcription factors [42]. Much insight on the roles of KLFs in the regulation of human developmental processes have been contributed by studies conducted on model organisms such as mice, zebrafish and flies. To be specific, KLF6 is expressed throughout embryonic and tissue development, including the development of kidneys [43], eyes [44], prostate [45] and several other tissue types [46], thus signifying its importance in developmental process regulation. Indeed, the homozygous mutant Klf6^−/−^ mice died at E12.5 due to defects in haematopoiesis and yolk sac vascularisation [47]. It was further demonstrated that KLF6 bi-allelic deletion in embryonic stem (ES) cells impaired the cell differentiation and proliferation capability [47]. Furthermore, KLF6 is involved in the expansion and maintenance of hematopoietic stem cells and progenitor cells, as well as in the development of endoderm-derived organs, including the liver [48,49].

There are also other studies that further supported the KLF6 key roles in regulating cellular differentiation. For example, Racca et al. demonstrated that KLF6 regulates trophoblast differentiation, a precursor step for placenta development, by transactivating the expression of β-chorionic gonadotropin and pregnancy-specific glycoprotein genes in this cell type [50]. Additionally, KLF6 has been shown to induce the differentiation of preadipocytes into adipocyte by repressing the expression of delta-like 1 (Dlk1) via its interaction with HDAC3 [25,51]. In the central nervous system, KLF6 acts as the downstream effector for the GP130-STAT3 axis to transactivate the expression of importin-a5, which is required for oligodendrocyte progenitor cells differentiation and neurons myelination [52]. KLF6 facilitates the adaptation of pancreatic β-cells to metabolic stress both by promoting and preventing β-cell proliferation and insulin resistance-induced dedifferentiation/transdifferentiation into glucagon-producing α-cells, respectively [53]. During skeletal myogenesis, KLF6 is a downstream target of the Smad3-dependent TGFβ signalling pathway in supporting myoblast proliferation and survival [54].

### 5.2. Immune and Inflammatory Responses

The macrophage is a part of the innate immune system that plays critical roles in modulating early immune and inflammatory responses during infections or tissue injuries [55]. The polarisation of macrophages either into M1 (proinflammatory) or M2 phenotypes (anti-inflammatory) rely on cues from the environments. These signals will be transduced intracellularly to the downstream effectors—normally, the transcription factors—which will subsequently regulate the expression of appropriate immune and inflammation responses-associated genes [56]. In-line with the key roles of transcription factors in modulating this process, a high expression of KLF6 was found in human and murine macrophages [57]. Functionally, KLF6 regulates macrophage polarisation to the M1 phenotype and the expression of proinflammatory genes by cooperating with NF-κβ and suppressing the expression peroxisome proliferator-activated receptor gamma (PPARγ) and B-cell lymphoma 6 (BCL6) [57,58]. In support of these findings, an independent study by Zhang et al. reported that KLF6 acted as a NF-κβ coactivator to regulate the expression of several NF-κβ downstream targets, including the proinflammatory cytokines monocyte chemoattractant protein-1 (MCP1), chemokine C-X-C motif ligand 2 (CXCL2) and interleukin 8 (IL-8) [59].

Interestingly, a recent study has unravelled a novel KLF6 role in regulating HIF1α expression and the hypoxic-response transcriptional program in macrophages [60]. The activation of this hypoxia-associated gene expression program would allow the macrophage to adapt and survive at the potentially hypoxic site of infection and injury and the subsequent elicitation of proinflammatory responses. Besides, some reports showed a direct interaction between KLF6 and miRNAs in regulating macrophage polarisation. Kim et al. demonstrated that KLF6 was able to upregulate the expression of proinflammatory genes in the macrophage by repressing the expression of anti-inflammatory miR-223 [61]. Additionally, an independent study by Bi et al. showed that the miR-181a-mediated suppression of KLF6 and CCAAT-enhancer-binding proteins (C/EBPα) promoted macrophage polarisation towards the anti-inflammatory M2 phenotype [62]. Collectively, these findings highlight the KLF6 involvement in modulating macrophage polarisation and the elicitation of proinflammatory responses.

Nitric oxide (NO), which is a free radical that is produced upon L-arginine oxidation by nitric oxide synthase (NOS), also contributes to the innate immunity in combating the invading pathogens [63]. Various cell types are able to release NO, including macrophages, neutrophils, vascular endothelial cells, neuronal cells and lung epithelial cells [63]. As an immune response-associated transcriptional regulator, KLF6 is also involved in regulating NO production by directly transactivating the expression of inducible nitric oxide synthase (iNOS) in response to environmental stimulants or challenges [64]. Two other NOS isoforms exist: i.e., neuronal NOS (nNOS) and endothelial NOS (eNOS). Subsequent findings showed that KLF6 was required for nitric oxide-mediated apoptosis of the infected cells during flu and respiratory syncytial virus infections [65,66]. It is important to highlight that excess NO production would lead to hyperinflammation and profound tissue damage, thus aggravating the severity of the infection and disease. Therefore, the activation of iNOS and downstream NO production must be precisely regulated by their upstream regulator—which, in this case, is the KLF6—in conferring protections against the invaders.

### 5.3. Tissue Injury and Wound Healing

The responses towards tissue injury and the promotion of wound healing involve coordinated crosstalk between the injured tissue and microenvironment that includes the immune cells, endothelial cells and extracellular matrix [67]. These processes are modulated by the pertinent growth factors and gene expression programs that are activated within the injured tissue, as well as its surroundings, thus ensuring that the appropriate post-injury cascades are undertaken [68]. TGFβ is among the growth factors that plays an essential role in modulating tissue repair and wound-healing processes [69]. TGFβ expression during tissue injury was shown to be directly transactivated by KLF6 [10]. Along with TGFβ, KLF6 also regulates the expression of several other members of the TGFβ pathway, including COL1A1 [11], PLAU [12] and ENG [13], further highlighting KLF6′s roles in governing the wound-healing processes. For example, KLF6 expression was upregulated in hepatic stellate cells (HSCs) during acute/chronic liver injury and was found to transcriptionally regulate the expression of COL1A1, an extracellular matrix protein that participates in the wound-healing process [11]. Of note, HSCs are a population of mesenchymal cells residing within the liver. Upon activation by signals from the damaged cells, HSCs will secrete extracellular matrix proteins and other factors that will facilitate the repair wound-healing processes [70]. Besides, KLF6 expression was also found to be elevated in acute liver failure patients and mice models. Functionally, KLF6 mediated the autophagy process, which was to clear the damaged cells prior to liver generation, via the direct transcriptional activation of autophagy-associated genes ATG7 and BECLIN1 [71].

**Figure 5:**
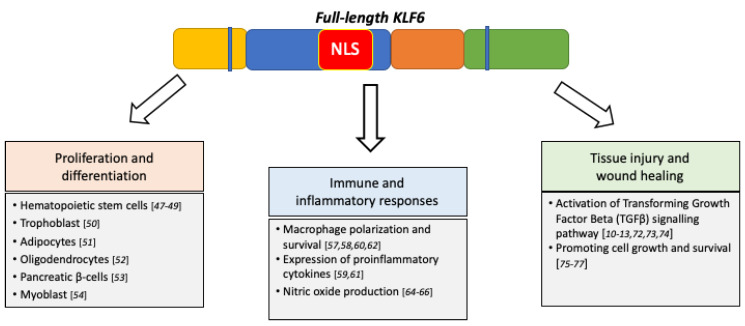
Diagram summarising the roles of KLF6 in normal physiological processes.

The roles of KLF6 in tissue repair and remodelling are not only limited in hepatocytes but, also, other cells types. KLF6 mediated the transactivation of vascular injury and repair-related genes such as PLAU, ENG, activin-receptor like kinase 1 (ALK1) and membrane metalloproteinase 14 (MMP14), of which ENG and ALK1 are members of the TGFβ signalling pathway [12,13,72,73]. In-line with these findings, the marked upregulation of KLF6 and TGFβ were also observed at the early phase of kidney ischemic reperfusion (I/R) injury, and KLF6 targeting impaired the apoptotic process following the ATP-induced ischemia [74]. Whilst KLF6 upregulation might induce apoptosis during I/R injury, KLF6 was found to be involved in promoting podocyte survival during injury. The deletion of podocyte-specific Klf6 in mice increased the susceptibility to Adriamycin-induced glomerulosclerosis and tubulointerstitial injury, followed by podocytes apoptosis [75]. It was demonstrated that KLF6 modulated the mitochondrial functions by preventing the activation of the cytochrome C-mediated intrinsic apoptotic pathway [75]. A subsequent study revealed that KLF6 was also involved in protecting the mitochondria from injury and apoptosis under diabetic conditions [76]. In the central nervous system (CNS), KLF6 interacted with STAT3 to coregulate the expression of genes involved in axon and corticospinal tract neurons regeneration following injury [77]. Overall, these findings denote KLF6 functions as the early responder to tissue injury and the mediator of tissue repair and wound-healing processes, in part by transcriptionally activating the expression of TGFβ and several other key components of the TGFβ signalling pathway. The roles of KLF6 in normal physiological processes are simplified in Figure 5.

## 6. KLF6 Implication in Human Diseases

The perturbation of normal physiological processes and homeostasis can contribute to the development of diseases. As discussed above, to prevent these processes from going awry, they are tightly regulated by coordinated interactions of the respective regulatory networks [78]. In this review, we will specifically focus on the roles of KLF6 in driving cancer and inflammatory-associated disease pathogenesis. Cancer is a group of diseases caused by uncontrolled cell growth, manifested by the cells’ capability to proliferate indefinitely, evade immunosurveillance and resist apoptosis [79]. Besides, inflammation also promotes cancer emergence and progression [79]. Since KLF6 plays a central role in modulating these processes, genetic alterations and/or the aberrant expression of KLF6 have been reported to support the formation and progression of many cancer types [17]. Furthermore, KLF6 has also been implicated in the pathogenesis of several inflammatory-associated diseases due to its key function as the mediator for proinflammatory and tissue injury responses. Thus, even a slight dysregulation in KLF6 functions could potentially perturb these processes, resulting in hyper-inflammation and tissue damage.

### 6.1. Cancer

#### 6.1.1. Full-Length KLF6

**Figure 6:**
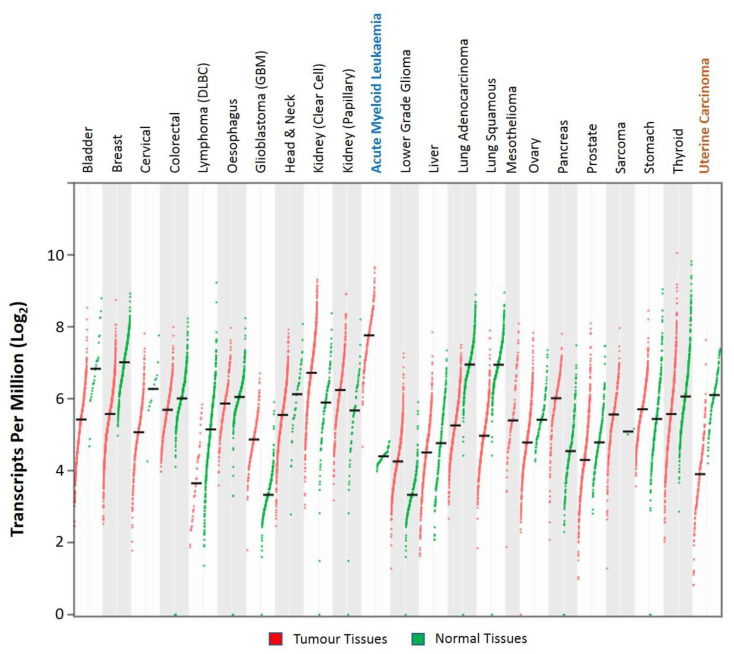
Dot plots representing the KLF6 expression RNA-Seq data in tumour and normal tissue samples from The Cancer Genome Atlas (TCGA) and The Genotype-Tissue Expression (GTEx) databases, respectively. KLF6 was found to be significantly upregulated and downregulated in acute myeloid leukaemia and uterine carcinoma, respectively (Log_2_FC |2|; *p*-value < 0.05). The data were analysed and visualised using the GEPIA2 web application [80].

Analysis of the publicly available large-scale The Cancer Genome Atlas (TCGA) RNA-Seq data showed that the expression of the KLF6 gene varied among cancer types (Figure 6). KLF6 was highly expressed in acute myeloid leukaemia, renal cancer of clear cell and papillary subtypes, glioblastoma multiforme and pancreatic cancer [80]. Apart from these, the expression of KLF6 was found to be downregulated in other cancer types as compared to their respective normal tissues [80]. Early reports showed that KLF6 was frequently inactivated in sporadic prostate cancer cases. Loss-of-heterozygosity (LOH) analysis revealed that KLF6 inactivation conformed to the Knudson’s two-hit hypothesis, thus classifying KLF6 as a tumour suppressor [81]. Stemming from this prostate finding, the KLF6 mutational status, expression level and tumour-suppressive roles were extensively studied in various cancer types. Consistent with the reports by Narla et al. [81], KLF6 was also found to be either inactivated or downregulated in a significant fraction of colorectal cancer [82], non-small cell lung cancer [83], ovarian cancer [15], gliomas [84] and hepatocellular carcinoma [85] cases. In addition, promoter hypermethylation and suppression by the noncoding RNAs and microenvironment factors could also result in KLF6 downregulation in cancer [86,87,88,89,90]. KLF6 functional interrogations revealed that KLF6 overexpression had negative effects on tumour growth and progression, whereby KLF6 silencing resulted in increased tumorigenicity [83,91,92,93,94]. Mechanistically, it has been demonstrated that KLF6 exerts its cell growth suppressive functions by modulating the expression of genes involved in the cell cycle, apoptosis and senescence regulation [81,95,96,97,98,99].

**Figure 7:**
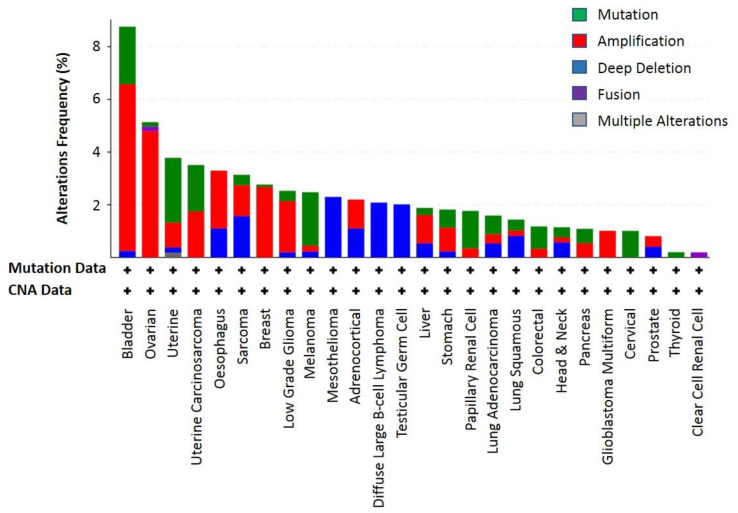
The frequency of KLF6 genetic alterations in twenty-six different cancer types that were molecularly characterised in the large scale TCGA project. These KLF6 genetic alterations frequency data were queried and extracted using cBioPortal (https://www.cbioportal.org/) [108,109]. The identified genetic alterations in KLF6 include mutation, amplification, deep deletion, fusion and multiple alterations. The “+” sign on top of each cancer type indicates that the mutation and copy number alteration (CNA) data are available for these cancer types.

Nonetheless, the KLF6 mutational status in cancer has been a subject of debate. Several independent studies have reported the absence of KLF6 genetic alterations in colorectal, liver, brain and prostate cancers [100,101,102,103,104,105,106], contradicting earlier studies that showed the frequent KLF6 inactivation in these cancer types. It has been pointed out that PCR amplification artefacts due to the use of archived cancer tissues or other technical issues might contribute to these discrepancies in the KLF6 mutational status [107]. Moreover, analyses of the more recent TCGA genomic data revealed that KLF6 was rarely deleted/inactivated in the previously studied cancers [108,109]. Collectively, the percentage of cancer cases that had the KLF6 gene alteration were quite low (Figure 7). Among the analysed cancer types, bladder cancer had the highest frequency of KLF6 genetic alterations—36 out of 411 cases—of which 2.19% were mutations, 6.33% were amplifications and 0.24% were deep deletions. These data indicate that KLF6 was not a common target for genetic alterations, and its deregulation in cancer could be caused by other mechanisms, potentially at the level of transcriptional or translational regulations.

Contrary to its widely reported role as a tumour suppressor, there were several studies that reported the growth-promoting functions of KLF6 in cancer. The pro-oncogenic fusion protein RUNX1-ETO upregulated the expression of KLF6 in acute myeloid leukaemia [21]. KLF6, which is highly expressed in AML, cooperated with RUNX1-ETO to drive the expression of this fusion protein downstream of targets in promoting leukaemia development [21]. Two independent studies by Sirach et al. and D’Astolfo et al. demonstrated that KLF6 protected hepatocellular carcinoma (HCC) cells from apoptosis to promote HCC progression [110,111]. Meanwhile, in ductal breast carcinoma, KLF6 colocalised with the ERBB2 oncoprotein in the nucleus, whereby its expression positively correlated with the estrogen receptor alpha expression [112]. Targeting KLF6 in the breast cancer cell line reduced the cells’ proliferative capacity, denoting KLF6 pro-oncogenic roles in breast cancer [112]. A recent study discovered that KLF6 expression in clear cell renal cell carcinoma (ccRCC) was driven by a super-enhancer that resulted in its high expression in ccRCC tissues and cell lines [113]. CRISPR-mediated KLF6 targeting impaired ccRCC cell growth both in vitro and in vivo due to perturbation in the mTORC1 signalling pathway and lipid homeostasis [113]. These findings were consistent with the growing evidence demonstrating that cancer cells are highly dependent on the super-enhancer-driven genes to support their development and progression [114,115].

#### 6.1.2. KLF6 Spliced Variants

The contradicting observations on the KLF6 mutational status and role in cancers have been further confounded by the discovery of KLF6 spliced variants and their involvement in regulating tumorigenesis, particularly KLF6-SV1. This KLF6 alternative splicing was induced by the G > A germline mutation at the intervening sequence 1-27, in which this mutation was first identified in prostate cancer cases, which lead to alternative splicing. This single nucleotide polymorphism was associated with an increased risk of developing prostate cancer [14]. The hepatic growth factor and RAS signalling pathway have also been linked to enhancing the KLF6 alternative splicing event in cancer [116,117]. Whilst the full-length KLF6 is established as a tumour suppressor in many cancer types, KLF6-SV1 is deemed to be pro-oncogenic by antagonising the full-length KLF6 functions in a dominant-negative manner. KLF6-SV1 has been reported to promote the progression and associate with a poor prognosis of the following cancer types: prostate [118,119], breast [120], ovarian [121] and lung cancer cells [122,123]. Intriguingly, KLF6-SV2, which also lacks the NLS domain, was downregulated in colorectal and liver cancers and possessed the tumour suppressive roles. The overexpression of KLF6-SV2 in colorectal and liver cancer cells reduced the cell proliferation whilst inducing apoptosis [124,125]. Even though the role of KLF6 and its SV1 and SV2 counterparts have garnered significant interests, KLF6-SV3 functions are yet to be elucidated. The functional roles of full-length KLF6 and the three KLF6 spliced variants in cancer are summarised in Table 1.

Nonetheless, the presence of the IVS 1-27 G > A germline mutation has also been controversial due to several opposing findings. This variant was detected at a significantly lower frequency in Ashkenazi prostate cancer patients as compared to the control subjects [126]. Similarly, a genome-wide association study in the Italian lung cancer patients cohort revealed that IVS 1-27 G > A was significantly associated with a reduced risk of lung cancer, whilst the validation study using the Norwegian lung cancer patients cohort showed no significant association between this polymorphism and the risk of developing lung cancer [127]. Similarly, there was no significant association between IVS 1-27 G > A and risk of developing benign prostate hyperplasia and prostate cancer in the Finnish population [128]. Hence, the functional impact of this polymorphism towards cancer susceptibility could vary between different populations or ethnic groups.

### 6.2. Metabolic and Inflammatory Diseases

Liver serves as the hub organ that governs and responds to the body’s metabolic and energy demands. Glucose, lipid and protein metabolisms take place in the liver, tightly controlled by metabolic-associated transcriptional networks in response to the hormonal cues, nutrient status and body-fed state [129]. Dysregulation in liver metabolic functions could induce the development of insulin resistance and dyslipidaemia, for instance, which subsequently promotes the pathogenesis of metabolic disorders such as non-alcoholic fatty liver disease (NAFLD), hypercholesterolemia and type II diabetes [129]. Specifically, NAFLD could advance to non-alcoholic liver steatohepatitis (NASH), which is marked by chronic liver inflammation and advanced fibrosis. Patients with NASH could develop life-threatening complications, including liver failure, liver cancer and cardiovascular diseases [130].

KLF6 has been shown as one of the key regulators in liver development and functions, particularly in response to injury and NAFLD pathogenesis. Functionally, KLF6 was shown to directly transactivate the expression of glucokinase, a glucose sensor that functions to catalyse the conversion of glucose to glucose-6-phosphate [131]. Targeting KLF6 reduced the glucokinase expression that consequently led to an increased hepatic insulin resistance and the development of NAFLD [131]. Additionally, KLF6 deletion, either hepatocyte-specific or in all tissues, improved the insulin sensitivity and rendered protection against high-fat diet-induced steatosis via the post-transcriptional regulation of PPARα and its downstream targets [132]. Interestingly, full-length KLF6 and KLF6-SV1 also had opposing roles in driving the progression of NAFLD, similar to those observed in cancer. A liver biopsy analysis of patients with NAFLD revealed that the elevated expression of full-length KLF6 was associated with advanced NAFLD, characterised by increased steatosis and fibrosis [133]. On the contrary, patients with KLF6 IVS 1-27 G > A polymorphism reported lower numbers of liver fibrosis, as well as a milder form of NAFLD [133]. The expressions of KLF6 and TGFβ were found to be augmented during the progression of NAFLD to NASH, thereby corroborating the roles of the KLF6-TGFβ axis in the development and progression of liver fibrosis and steatosis [134].

Epithelial-to-mesenchymal transition (EMT) plays a pivotal role in tissue repair and the development of fibrosis. Two independent studies identified the TGFβ-KLF6 axis as playing a central role in driving renal and pulmonary fibrosis in the in-vitro and in-vivo models of diabetic nephropathy [135,136]. The anti-inflammatory protein PPARγ was found to be one of the downstream targets of the TGFβ-KLF6 pathway during renal inflammation and the progression of diabetic nephropathy. KLF6 negatively regulated the expression of PPARγ that, consequently, led to the de-repression of proinflammatory macrophage inflammatory protein-3 alpha (MIP-3), a PPARγ target gene [137,138]. In addition to its role as the mediator of diabetic-associated complications, KLF6 has also been implicated in promoting the pathogenesis of mesangial proliferation glomerulonephritis (MsPGN). It has been demonstrated that the lysine acetyltransferase 7 (KAT7)-mediated acetylation of KLF6 directly transactivated the expression of chemokines MCP1 and RANTES, subsequently leading to the proliferation and inflammation of glomerular mesangial cells, extracellular matrix accumulation and development of proteinuria [139].

An earlier finding by Tarabishi and colleagues showed that KLF6 and TGFβ were among the initial genes to be upregulated following ischemic reperfusion injury [74]. A recent study by Zhang et al. found that KLF6 was involved in coactivating the NF-κβ-mediated inflammatory response that, in turn, exacerbated the severity of the ischemic-reperfusion injury [140]. Meanwhile, they also reported that miR-181d-5p conferred a protective role against renal ischemic injury by reducing inflammation and cells death via its inhibitory role on KLF6 expression [140]. Besides the aforementioned diseases and complications, KLF6 has also been shown to contribute to the pathogenesis of inflammatory bowel (IBD), pulmonary vascular and muscle degenerative diseases [141,142,143]. In regard to IBD, KF6 expression was significantly upregulated in the IBD clinical samples and animal model, characterised by chronic intestinal inflammation due to macrophage polarisation from inflammatory anergic towards the pathogenic M1 phenotype [141]. Consistent with previous reports, KLF6 promoted IBD progression by cooperating with the proinflammatory-associated NF-κβ signalling pathway whilst suppressing the STAT3 anti-inflammatory signalling pathway in macrophages [141].

## 7. KLF6 Therapeutic Potential

The roles of KLF6 in cancer—particularly, the full-length KLF6—has been the subject of active investigations and discussions. This is due to the contradicting reports on the full-length KLF6 roles and mutations status in a myriad of studies, as well as variations in the KLF6 gene expression level in different cancer types. Nonetheless, shreds of evidence from functional studies have demonstrated that full-length KLF6 possesses growth-suppressive roles in several cancers, such as prostate, ovarian, colorectal and liver. Since KLF6 was found to be either downregulated or inactivated in these cancer types, direct KLF6 targeting for cancer therapeutic purposes might not be a viable option. Thus, one possibility is to reintroduce or upregulate the expression of tumour-suppressive full-length KLF6 in these cancer types. This could potentially be achieved by targeting the specific upstream regulators that mediate the full-length KLF6 repression or inactivation in cancers such as the miRNAs and long noncoding RNAs [87,88,144]. For example, KLF6 is the downstream target of miR-342-p in pancreatic cancer cells, whereby stable expression of KLF6 reduced the cancer cells’ viability and increased the sensitivity to gemcitabine [144]. Besides, the treatment with lovastatin, a hypercholesterolemia drug, was also able to induce the expression of KLF6 in cisplatin-resistant HCP4 cervical cancer and PCDP5 prostate cancer cells, leading to cell cycle arrest and apoptosis [145].

On the other hand, direct KLF6 targeting might be more relevant and applicable to the cancer types where KLF6 functions as pro-oncogene. Even though transcription factors, if not all, are deemed to be undruggable due to the lack of domains that can be chemically targeted, there has been a line of evidence demonstrating that transcription factors can indeed be targeted by small molecule inhibitors. One example of this is the successful development of small molecule inhibitors targeting hypoxia inducible factor 2 alpha (HIF2α) in renal cell carcinoma [146,147]. Alternatively, the efforts to develop novel anticancer therapy could be centred on targeting the pro-oncogenic KLF6-SV1 isoform. In cancers where pro-oncogenic KLF6-SV1 supports tumorigenesis, primarily by antagonising the tumour-suppressor full-length KLF6, depleting this KLF6-SV1 isoform seems an attractive option. Indeed, several studies have shown that inhibiting this cytoplasmic-localised KLF6-SV1 was effective in suppressing tumour growth and progression both in vitro and in vivo [121,122,148]. Furthermore, there are functional modulators that can inhibit alternative splicing [149]. Therefore, it is interesting to explore whether this alternative splicing inhibitor could reduce the expression of the pro-oncogenic KLF6-SV1 whilst maintaining the expression of full-length KLF6 [149].

Due to the KLF6 key roles in promoting the pathogenesis of several metabolic and inflammatory diseases, KLF6 could also be a therapeutic target candidate for these diseases. However, direct KLF6 targeting KLF6 might be complicated, because KLF6 also participates in the regulation of normal physiological processes [52,57,72,75]. If novel therapeutic strategies for these diseases are to be developed around KLF6, the ideal avenue forward would be to target the KLF6-regulated downstream pathways. Despite the above speculations on the KLF6 therapeutic potential, it might still be a while before KLF6 could eventually be developed into an efficient therapeutic target and reach the bedside. This is largely due to the complexity and limited understanding on the biology and roles of this transcription factor in both physiological and pathophysiological settings. Hence, it is vital to first increase our understanding on the KLF6 biology and address the possible reasons behind the contradicting observations on KLF6 functional roles, in which these should be the main priority for the future KLF6 studies.

## 8. Future Perspectives and Concluding Remarks

In sum, KLF6 plays key roles in regulating cellular proliferation and differentiation and modulating inflammation and immune responses, as well as promoting tissue repair and wound healing. It is important to highlight that KLF6 may have different functional roles in different tissue types or physiological processes. Several factors might contribute to KLF6 functional diversities. First, KLF6 may co-opt or interact differently with the existing regulators and gene expression programs of a particular tissue type or biological processes. Next, the tissue-type or context-specific epigenetic regulations may also play roles in controlling the KLF6 expression level and functions. For instance, in a particular cell type or physiological setting, the chromatin structure spanning the KLF6 and/or its downstream target genes loci are more “relaxed”, which would, in turn, be accessible by transcriptional machineries, leading to KLF6 upregulation and the subsequent activation of its downstream molecular networks. In-line with this, the dynamic of the chromatin landscape can also change in response to the relevant intrinsic and/or extrinsic cues. For example, during tissue injury, the damaged cells might release signals that would modify the chromatin landscape, resulting in the expression of KLF6 and the associated tissue repair mechanism-associated genes. In this regard, it is worth it to further investigate on how the interactions between KLF6 and different molecular players in different biological contexts would contribute to KLF6 functional diversity.

In the cancer setting, the actual role of KLF6 is yet to be completely established due to the numerous contradicting findings. Some studies demonstrated that KLF6 functions as a tumour suppressor, while others showed that KLF6 promotes tumour growth. These KLF6 dual functions could be due to the KLF6 interactions with different driver mutations, tissue-specific gene expression programs and epigenetic regulators, as well as other related factors in particular cancer types or stages. To address this, future studies on KLF6 should also focus on elucidating the cancer type-specific interactome of KLF6 and how this interactome may modulate the KLF6 functions in cancer and the outcomes of cancer progression in general. Moreover, the presence of KLF6-spliced variants has added another layer of complexity in the effort understanding the roles of KLF6 in cancer. Hence, it is vital in future studies to first confirm the predominantly expressed KLF6 isoforms, whether the full-length KLF6 or any of the three spliced variants. In addition, it is also useful to revisit the large-scale TCGA RNA-Seq data to determine whether the TCGA findings support the reported presence of these spliced isoforms. If it confirms, then the targeting constructs in any knockdown/knockout experiments should be designed to exclusively target the isoforms of interest. This would avoid any confounding effects and ensure that the phenotypic outcomes can be linked to the correct isoforms.

Meanwhile, it remains poorly understood how the IVS 1-27 G > A polymorphism can give rise to three splicing isoforms that utilise different splicing donor and/or acceptor sites. Therefore, it is worth investigating how the cells dictate which isoform should be expressed in the presence of this polymorphism and the molecular mechanisms that are involved in driving this process. One exciting approach to tackle this question is to utilise the latest CRISPR-mediated base-editing and prime-editing tools to introduce this specific SNP in the in-vitro model [150,151], followed by interrogating the ability of this SNP to induce alternative splicing, as well as other molecular players that are involved in regulating this process. Overall, the presence, mechanisms of regulation and functions of these spliced variants must be carefully studied and addressed. This knowledge holds the key to better understanding the biology of KLF6 in general and, importantly, shedding light on its enigmatic roles in both physiological and pathophysiological settings.

## Figures and Tables

**Table 1:** The functional roles of full-length KLF6 and KLF6 spliced variants in cancer.

Isoforms	Functions
Full-length KLF6	Downregulated expression in cancers, except in acute myeloid leukaemia, kidney (clear cell and papillary subtypes), glioblastoma multiforme and pancreatic cancer (TCGA RNA-Seq). Widely regarded as a tumour suppressor [91,92,93,94,95,96,97,98,99], despite the contradicting findings showing full-length KLF6 has a growth-promoting role [21,110,111,112,113].
KLF6-SV1	Pro-oncogenic by promoting the progression and association with poor prognosis in prostate [118,119], breast [120], ovarian [121] and lung cancers [122,123]. Antagonising the growth-suppressive properties of KLF6 in a dominant-negative manner.
KLF6-SV2	Downregulated in liver [124] and colorectal cancers [125]. Has antiproliferative and proapoptotic functions in colorectal and liver cancers.
KLF6-SV3	Functions are yet to be elucidated.
